# Exploring the Impact of Human–Animal Connections and Trust in Labeling Consumers’ Intentions to Buy Cage-Free Eggs: Findings from Taiwan

**DOI:** 10.3390/foods12173310

**Published:** 2023-09-02

**Authors:** Min-Yen Chang, Ching-Tzu Chao, Han-Shen Chen

**Affiliations:** 1 Department of Accounting, Jiaxing University, Jiaxing 314001, China; mingyen0223@zjxu.edu.cn; 2 Department of Health Industry Technology Management, Chung Shan Medical University, Taichung 40201, Taiwan; tiffany891224@gmail.com; 3 Department of Medical Management, Chung Shan Medical University Hospital, Taichung 40201, Taiwan

**Keywords:** sustainable development goals (SDGs), value-attitude-behavior model, food transformation, food choice, animal welfare-friendly products

## Abstract

Recently, interest in animal welfare has steadily increased, driven by a growing focus on environmental, social, and governance (ESG) issues. This trend aligns with the Sustainable Development Goals (SDGs) set by the United Nations. This underscores the importance of comprehending consumer attitudes towards products that are respectful to animal welfare. This research aims to employ the Value-Attitude-Behavior (VAB) model as a theoretical basis to explore the behavioral intentions of Taiwanese consumers to buy cage-free eggs. To provide comprehensive insights into consumer behavior in this domain, the study examines key variables such as ‘human–nature connectedness’, ‘trust in labels’, and ‘attitude towards animal welfare’. By addressing an existing research gap in the literature and exploring consumer behavior in relation to animal welfare-friendly products, the research contributes to an area that has received limited attention. We gathered a total of 341 valid questionnaires for this research. Employing Structural Equation Modeling (SEM) along with Maximum Likelihood Estimation (MLE), we investigated the causal connections between variables. The study presents three key observations: First, consumers who value sustainability and ethics tend to maintain a positive perspective on free-range eggs. Second, a favorable stance towards cage-free eggs directly correlates with positive future behaviors. Lastly, label trustworthiness and a supportive view towards animal welfare heavily affect behavioral intentions in consumers. Given these insights and the growing significance of animal welfare in consumer choices, we recommend that participants in the food and livestock sector earnestly advocate for and back practices that prioritize animal well-being.

## 1. Introduction

Unsustainable consumption and production practices significantly contribute to climate change, biodiversity loss, and environmental pollution [1]. Methane emissions, particularly from agricultural activities associated with ruminant livestock, play a substantial role in these issues [2]. Excessive consumption of poultry and livestock products has adverse consequences for human health and the environment, especially due to intensive and large-scale industrial farming practices that negatively impact animal welfare. Reducing the livestock population and promoting animal-friendly practices are effective strategies for mitigating greenhouse gas effects.

In 2015, the United Nations (UN) revealed the Sustainable Development Goals (SDGs), a framework designed to steer global endeavors towards sustainable development. Recognizing the significance of animal welfare in achieving the Sustainable Development Goals, the United Nations Environment Assembly [3] passed a resolution in 2022. This resolution merges animal wellbeing considerations into four key goals. The objectives consist of Goal 12, focusing on sustainable consumption and production. Goal 13 is dedicated to climate action, Goal 14 pertains to marine life, and Goal 15 is concerned with land-based biodiversity. This emphasizes the significance of including animal welfare-friendly measures in the practices, corporate conduct, and trade activities of international organizations to promote sustainable development.

Growing concerns about environmental sustainability and animal welfare have sparked increased awareness of the importance of animal-friendly farming practices [4]. Consumers now consider the welfare of animals when making purchasing decisions regarding poultry and livestock products. Animal welfare encompasses multiple stages, including rearing, slaughtering, and transportation, aiming to ensure the well-being and fundamental freedoms of animals [5,6]. This focus on animal welfare is evident in the European Union’s codes of conduct and sustainability reporting systems [7,8,9], as well as Taiwan’s Council of Agriculture’s publication of the “Animal Welfare White Paper” as a guiding framework for creating an animal-friendly society.

**Figure 1:**
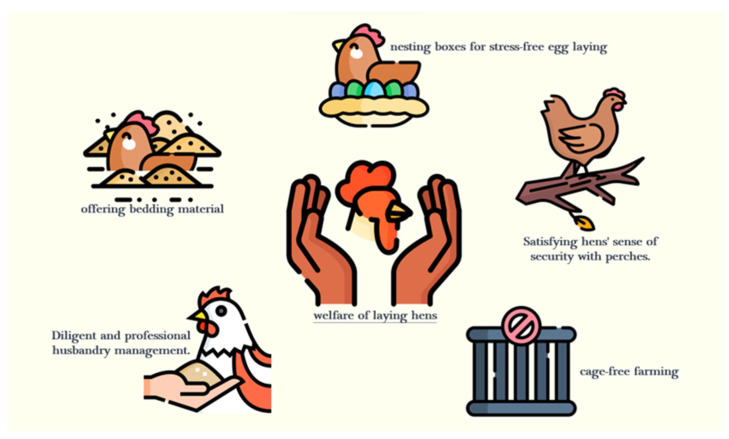
The research background.

In response to consumer preferences, companies, such as McDonald’s in the UK and Carrefour Taiwan, have started offering animal welfare-friendly products. Studies indicate that customers care about the well-being of farm animals and are ready to pay a premium for products that prioritize this aspect [10]. Notably, data from Taiwan’s Ministry of Agriculture (MOA) [11] reveal a substantial demand for eggs, with an average annual consumption of 356 eggs per person, ranking the country second globally after Mexico. Therefore, this study focuses primarily on cage-free eggs as a crucial element of animal welfare-friendly products (see Figure 1 for the research background).

Yang [12] found that egg producers expressed a negative attitude toward cage-free systems (CFS) due to concerns about hen health, reduced efficiency, and profitability. Obstacles to transitioning to CFS were identified, including consumer unwillingness to pay, lack of reliable certifications and labeling, limited land availability, environmental protection policies, and CFS management challenges. Another study by Cao et al. [13] suggested that consumers rely on personal experiences and buying habits when making egg purchases. Consumers with limited experience may be more receptive to information about animal welfare, thus presenting an opportunity to promote the purchase of animal welfare eggs.

To examine consumer behavioral intentions towards animal-friendly products, our study employed the widely used Value-Attitude-Behavior (VAB) model in consumer behavior research. This model underscores the impact of values on attitudes and behavior, as highlighted by Cheung et al. [14] and Chryssohoidis et al. [15]. The objective was to assess whether consumers’ values towards animal welfare translate into positive attitudes and actual purchase behavior towards animal-friendly products. Previous research integrating moral cognition within the VAB model has examined how environmental factors, including the environment and animal welfare, shape consumers’ moral values and influence their choices of organic food [16].

Human–nature connectedness refers to individuals’ perception of the relationship between humanity and the natural environment, characterized by equality, mutual benefit, and harmony [17]. Direct interaction with the natural world significantly influences people’s attitudes and behaviors towards the environment, including aspects related to human health, well-being, and biodiversity [18]. As environmental awareness increases, individuals’ responsibility for environmental conservation and their consumption behavior towards environmentally friendly products have become important considerations. Past research has emphasized the growing trend among consumers to choose environmentally friendly products [19,20], focusing on the various ways in which people value environmental friendliness—a natural bond that has been scrutinized in studies.

The European Commission [21] conducted a survey revealing the significant importance that EU citizens place on improving Farm Animal Welfare (FAW). In response to this concern, impartial third-party organizations have developed stringent standards, known as “animal welfare certification”. These standards are utilized for auditing and certifying purposes, encouraging businesses to enhance animal welfare on livestock farms and ensuring compassionate treatment of animals. Certified labels enable consumers to identify and choose products that prioritize animal welfare, ensuring both animal well-being and food safety. Internationally recognized certified labels include “RSPCA Assured” in the UK, “Beter Leven” in the Netherlands, and “Animal Welfare Approved” in the US. In Taiwan, certified labels include “Humane Monitoring”, “Animal-Friendly Livestock Production”, “Animal-friendly Production Certification”, and “CFA”. Research has shown that consumers highly value certified labels when purchasing organic products [22] and exhibit higher levels of trust and willingness to pay when certification is conducted by impartial third-party entities [23]. Therefore, this study incorporates trust in labels as a research variable.

Animal welfare is gaining increasing significance in contemporary society, with individual attitudes and potential behavioral changes playing a crucial role in promoting and enhancing the lives of animals [24]. The connection between meat production and negative emotions associated with animal slaughter has been highlighted, particularly among vegetarians and strict vegans [25]. Thus, this study examines participants’ dietary habits to analyze their behavioral intentions towards animal welfare-friendly products. Furthermore, emphasizing the inclusion of pet ownership in population surveys highlights the perception of pets as integral members of the family unit [26]. Moreover, cultural and religious beliefs influence attitudes towards animal treatment [27,28], prompting this study to consider participants’ religious beliefs in exploring variations in behavioral intentions towards animal welfare-friendly products.

There is a significant research gap in understanding the factors that influence consumers’ intention towards free-range eggs. This gap presents an opportunity for further exploration. Therefore, this study operates on the framework of the VAB model, combining the three key research variables of “connection with nature”, “trust in labels”, and “attitude towards animal welfare”. By constructing a comprehensive model, this research aims to fill the research gap and gain a deeper understanding of consumer behavior regarding animal-friendly consumption, specifically focusing on free-range eggs. This study’s findings will offer a crucial understanding of consumer patterns, carrying practical significance for those overseeing the food and livestock sectors. Understanding the determinants of consumers’ behavioral intentions will enable managers to tailor marketing strategies, driving positive changes in consumer behavior and advancing animal-friendly practices in the industry. This research holds the promise of having a substantial, practical impact in its domain.

## 2. Reviewing the Literature and Developing Hypotheses

### 2.1. VAB Model

Values are fundamental beliefs that guide the actions and decisions of individuals. They are subjective in nature and influenced by social and psychological factors in a person’s development. Attitudes, on the other hand, represent individuals’ feelings and opinions, whether positive or negative, towards specific behaviors or objects. Attitudes have been widely recognized as important predictors of behavioral intentions [29] and as influential in shaping individual behavior [30].

The current study employs the VAB model, incorporating theoretical variables such as green values, attitudes towards animal-friendly products, and behavioral intentions. Each of these variables will be further explained in detail throughout the study.

#### 2.1.1. Attitude towards Cage-Free Eggs Driven by Values

Chen and Chang’s [31] study indicated that consumers generally form more favorable impressions when they perceive that a product has a greater emphasis on environmental sustainability or animal welfare. This finding is supported by the research of Ma and Chang [32]. Han et al. [33] also found that favorable attitudes and purchase behaviors arise when a product’s perceived green value aligns with consumers’ expectations.

Furthermore, Orellano et al. [34] noted that consumers’ religious attendance can inspire ethical consumption. Qasim et al. [35] underscored how an individual’s moral self-identity can harmonize their attitudes towards environmental conservation and animal welfare. According to Khalid et al. [36], individuals who believe in morally just actions based on universal principles tend to adopt favorable attitudes and show a strong concern for animal welfare, with the expectation of achieving desirable outcomes. Building on the existing literature, this study puts forth the following hypotheses:

**Hypothesis** **(H1):** *The values of consumers positively impact their perspectives on cage-free eggs*.

#### 2.1.2. Relation between Attitude towards Cage-Free Eggs and Behavioral Intentions

Consumer attitude consists of three elements: cognition, which involves understanding the subject; affect, which symbolizes the emotional assessment of the subject; and intention, which indicates anticipated future behaviors related to the subject [37]. Consumers’ attitudes towards environmental issues and ecological socio-benefits significantly influence their green purchasing behavior [14]. Amoako et al. [38] discovered significant positive correlations between consumers’ green knowledge, green attitudes, and purchasing behavior. It has been observed that promoting green values, offering behavioral choices, and providing recommendations can effectively enhance behavioral intentions towards green products [39]. Notably, consumers display a preference for products that are environmentally friendly and recyclable, as they seek to avoid harming the environment [40]. Building upon these insights, this study formulates the following hypothesis:

**Hypothesis** **(H2):** *Consumers’ attitudes towards cage-free eggs have a positive influence on their behavioral intentions*.

### 2.2. Human–Nature Connectedness

Enhancing human–nature connectedness through engagement with the natural environment and mindfulness practices has been recognized as a valuable approach for deepening individual understanding and experiencing the interdependence between human well-being and nature conservation [41,42]. Barragan-Jason et al. [43] demonstrated a positive correlation between human–nature connectedness and improved ecological sustainability, values, and pro-environmental behaviors. They also found a negative correlation with non-environmental or anti-environmental values. Similarly, Cheng et al. [44] identified a stronger inclination among individuals who possess a heightened sense of connection between humans and nature to support efforts towards environmental conservation. Park and Lin [45] observed that self-expression and environmental concerns indirectly influence purchase intentions. Additionally, the push for a healthier lifestyle and a growing concern for the environment have emerged as key influences on how consumers perceive food quality and determine their food purchasing decisions [46].

In summary, the literature suggests a correlation between human–nature connectedness and behavioral intentions. Drawing from this research, the study proposes the following hypothesis:

**Hypothesis** **(H3):** *Consumers’ sense of human–nature connectedness positively influences their behavioral intentions*.

### 2.3. Trust in Labels

Consumers’ decision-making processes are significantly influenced by trust, especially regarding food purchases [47]. Ngo et al. [48] found that consumer trust stimulates positive attitudes, which in turn increases purchase intentions. Certified labels serve as a mechanism to reduce uncertainty when evaluating product quality before making a purchase decision. By providing information about the production process, certified labels help consumers overcome uncertainties and facilitate their choices and purchase decisions [49]. Consumers tend to exhibit greater trust and purchase intentions towards products with government certifications [50]. Previous research has demonstrated that consumer trust in organic-certified labels significantly influences their willingness to pay, more so than in non-certified labels [51]. Moruzzo et al. [52] observed a clear consumer interest in organic-certified foods, particularly staple foods, such as rice, or commonly consumed items such as eggs. Drawing upon the literature reviewed, this study postulates the following hypotheses:

**Hypothesis** **(H4):** *Consumers’ trust in cage-free eggs with certified labels positively influences their behavioral intentions*.

### 2.4. Attitude towards Animal Welfare

While the primary goal of food consumption practices may not be to improve FAW, it is widely acknowledged that these practices have significant social repercussions for animal life [53]. Studies consistently show that concerns about animal welfare are more influential in the choice to purchase organic meat than other factors, such as health benefits, authenticity, and environmental conservation [54]. Consumer interest in animal welfare is steadily increasing, surpassing other factors related to food quality. Consumers view animal-friendly products as healthier, more delicious, hygienic, safe, acceptable, authentic, environmentally friendly, and traditional [4].

Furthermore, studies indicate that when assured of high-quality animal welfare, numerous individuals are willing to pay extra for animal-derived products. In fact, a considerable fraction is even willing to pay more than 5% extra [55]. Fernandes et al. [56] argued that there is a growing misalignment between the animal industry and societal expectations regarding animal welfare. Implementing measures to improve FAW can lead to societal benefits beyond production profits. Drawing upon the literature reviewed, this study puts forth the following hypothesis:

**Hypothesis** **(H5):** *The consideration of animal welfare positively influences consumers’ behavioral intentions*.

## 3. Supplies and Procedures

### 3.1. Framework of Research

**Figure 2:**
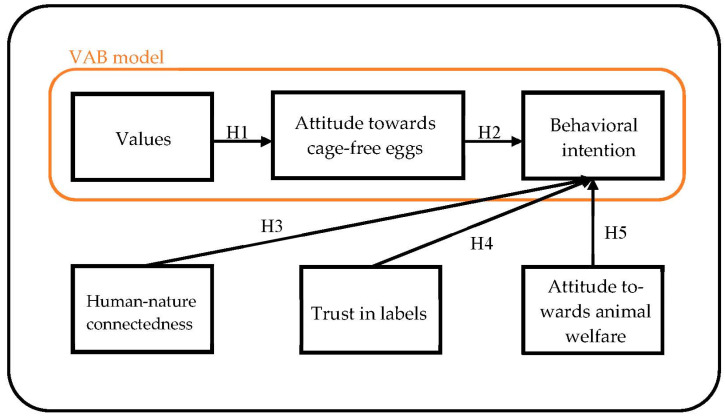
Conceptual framework and hypotheses.

This study utilized the VAB model as its primary structure and incorporated three study variables, namely human–nature connectedness, trust in labels, and attitude towards animal welfare, to examine the behavioral intentions of Taiwanese consumers regarding eggs produced under animal welfare-friendly conditions. The research framework is depicted in Figure 2.

### 3.2. Survey Design

The questionnaire design consisted of seven sections, each addressing specific aspects of the research topic. In the first section, seven questions were developed to examine values, integrating green values derived from Ma and Chang’s [32] study and moral values adapted from Agag and Colmekcioglu’s [57] study. The second section, comprising three questions adapted from Jung et al.’s [58] study, focused on exploring participants’ attitudes towards cage-free eggs. Section three, based on Zhao et al.’s [17] study, employed three questions to assess human–nature connectedness. The fourth section, designed to explore trust in labels, incorporated four questions derived from research conducted by My et al. [59] and Wang et al. [60]. Following that, the fifth section contained seven questions intended to probe participants’ perspectives on animal welfare, guided by the study of Carnovale et al. [55]. In the sixth section, four questions adapted from Nguyen et al.’s [61] study were designed to examine participants’ behavioral intention. The seventh and final section consists of eight questions designed to collect basic demographic data from the participants. This includes their gender, age, education level, personal monthly income, occupation, pet ownership, dietary preferences, and religious affiliations.

To ensure consistency in measuring responses, we utilized a 7-point Likert scale for all queries, extending from “strongly disagree (1)” to “strongly agree (7)”, the sole exception being the demographic section. Higher scores on the Likert scale mean a stronger positive evaluation of the attributes under consideration. This comprehensive questionnaire design allows for a thorough investigation of the research topic while collecting valuable insights from the participants.

### 3.3. Data Sampling and Collection

The widespread use of the Internet has led researchers to shift from paper-based surveys to online platforms for data collection. Online surveys offer advantages such as improved data completeness and resource efficiency [62]. A pilot test was conducted before distributing the official questionnaires, resulting in 64 valid responses. From 2 April to 9 May 2023, diverse methods, including word-of-mouth, personal Facebook, Instagram, Line groups, and other social media channels, were used to disseminate the questionnaires to individuals who had consumed or purchased cage-free eggs. Participant privacy was protected by ensuring clear communication about the study’s purpose and the anonymous nature of data collection. As the study was non-anonymous, non-interactive, and non-interventional without gathering identifiable information, it did not require prior ethical review. The research followed ethical guidelines, respecting participant privacy and confidentiality.

We gathered 421 questionnaires in total. After discarding the invalid ones, we were left with a final, valid sample of 341 participants. The sample exhibited a sampling bias towards female respondents, which aligns with the common characteristic found in consumer research. Women often outnumber men in food-related consumer research, possibly due to their primary responsibility for food purchases within households [63]. Refer to Table 1 for the breakdown of the sample’s demographic distribution.

### 3.4. Methods for Analyzing Data

This research relied on a quantitative methodology using a questionnaire survey to collect data. We meticulously analyzed the gathered information with two robust software tools, IBM SPSS Statistics 27.0 and AMOS 28.0. Statistical analysis techniques include descriptive statistics, such as frequency distribution, percentages, averages, and standard deviations. Additionally, we performed reliability and validity analyses to verify the precision of our measurements. We utilized SEM to probe causal dynamics within our proposed framework and to evaluate how well the overall model fits. The maximum likelihood estimation (MLE) technique was applied for this purpose. By employing these advanced analytical techniques, this study yielded compelling evidence to validate the proposed research hypotheses.

## 4. Evaluation and Findings

### 4.1. Assessing Models: Gauging Reliability and Validity

Table 2 presents the findings of the reliability and validity assessments for the various dimensions. As per Nunnally’s suggestion [64], a Cronbach’s α value exceeding 0.7 signifies high reliability. Conversely, values that fall between 0.35 and 0.7 denote moderate reliability, with anything below 0.35 indicating low reliability. In this research, every Cronbach’s α value in Table 2 surpassed 0.7, thereby implying that the measurement model demonstrates a high level of reliability.

Fornell and Larcker [65] established certain benchmarks for determining convergent validity. They proposed that the factor loadings’ standardization should be above 0.5, and the average variance extracted (AVE) should also exceed 0.5. Additionally, the composite reliability (CR) should surpass 0.6. Bagozzi and Yi [66] further suggested that if a CR value exceeds 0.60, it denotes robust internal consistency. In this study, all the factor loadings have surpassed the 0.5 threshold. Similarly, both the AVE and CR values comfortably exceeded their respective benchmarks of 0.5 and 0.6. These results point to a satisfying convergent validity and robust internal consistency of the adopted measurement model (please refer to Table 2).

Table 3 presents the correlation coefficients of our study’s measurement model alongside the square root of the Average Variance Extracted (AVE). We employed Fornell and Larcker’s [65] method, which suggests assessing discriminant validity by comparing the AVE’s square root with the correlation coefficients of the individual variables. In order for the validity to be considered strong, the AVE’s square root should surpass the correlation coefficients among the variables. In our research, all of the variables’ correlation coefficients were lower than the AVE’s square root, as illustrated in Table 3. This indicates that our variables satisfy Fornell and Larcker’s [65] conditions for discriminant validity in the measurement model.

### 4.2. Model Fitness Test

Our examination of the proposed hypotheses was conducted using the MLE method. The ratio of x^2^/df was 2.675, which implies a fairly good model fit. Furthermore, the values of NFI, CFI, and IFI were 0.925, 0.952, and 0.952, respectively, suggesting an acceptable fit. The PNFI value was 0.786, which met the standard criteria. Moreover, the RMR, SRMR, and RMSEA values were 0.077, 0.0382, and 0.070, respectively, all falling within the acceptable range. Although the GFI (0.871) and AGFI (0.835) values were slightly below the standard criteria, Bagozzi and Yi [66] suggested that a GFI above 0.90 is a conservative threshold, and a value exceeding 0.80 is considered acceptable. In light of most fitness indices satisfying or surpassing set benchmarks, the model from this investigation exhibited a powerful correlation with the data, thus demonstrating its superior conformity.

### 4.3. Model Path Analysis

**Figure 3:**
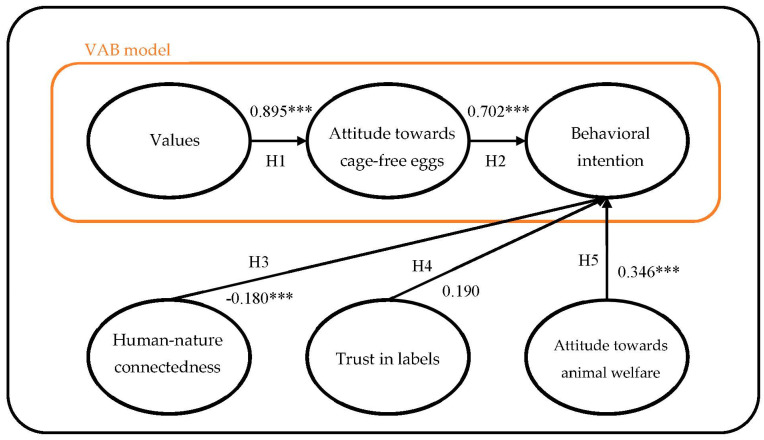
Model path analysis. Note: *** *p*-value is under 0.001.

Through SEM, this study explored the relationships between various variables, as detailed in Figure 3. The initial hypothesis (H1) demonstrated a notable positive correlation between values and attitudes regarding cage-free eggs (β = 0.736, *p* < 0.001). The second hypothesis (H2) proposed a comparably robust positive connection between attitudes towards cage-free eggs and intended behavior (β = 0.764, *p* < 0.001). On the contrary, Hypothesis H3 revealed a significant negative association between human–nature connectedness and intended behavior (β = 0.389, *p* < 0.001). While Hypothesis H4 proposed that trust in labels positively affects intended behavior, the result was not statistically significant (β = 0.697, *p* = 0.006). Ultimately, Hypothesis H5 asserted a considerable positive correlation between attitudes towards animal welfare and intended behavior (β = 0.711, *p* < 0.001).

The findings from this study validate Hypotheses H1, H2, and H5, as each of these hypotheses generated statistically significant outcomes. Hypothesis H4, although supported, did not reach statistical significance, while Hypothesis H3 was not supported. The detailed outcomes of the path analysis and hypothesis testing are shown in Table 4.

## 5. Discussion

The research results affirm Hypotheses H1 and H2, demonstrating that consumers’ green and ethical values considerably influence their positive attitude towards cage-free eggs. This is consistent with previous studies conducted by Ma and Chang [32] and Khalid et al. [36]. Additionally, this study revealed that a favorable attitude towards animal welfare-friendly products significantly influenced consumers’ intention to purchase cage-free eggs, consistent with the research results presented by Cheung et al. [14]. Spartano and Grasso’s [67] study also supported these findings, showing that consumers’ attitudes towards feeding hens with insects were influenced by environmental benefits, animal welfare considerations, and reduced food waste.

In contrast to expectations, Hypothesis H3, which proposed a positive influence of human–nature connectedness on consumers’ behavioral intentions, was not supported by the study’s findings. The findings greatly differ from the bulk of research done by Cheng et al. [44], Park and Lin [45], and Petrescu et al. [46]. This study suggests that although individuals may possess the concept of human–nature connectedness, it does not directly translate into their behavioral intentions. This observation aligns with Jin et al.’s [68] study, indicating that the importance consumers put on environmental conservation does not influence their buying decisions.

On the other hand, the research validates Hypothesis H4, which examines how trust in labels affects consumers’ decisions to buy cage-free eggs. The results of this research align with previous studies carried out by Lăzăroiu et al. [50], and Lang and Rodrigues [51]. Liu et al. [69] found that consumers in Chongqing, China, were willing to pay a significant increase in price for eggs labeled “organic”, “free-range”, or “nutrient-enriched”. This insight emerged from their research into the consumer inclination to spend more on eco-labeled eggs. Although our study differs in terms of examining animal welfare certifications, H4 received support, albeit without statistical significance. This discrepancy could be due to Taiwanese consumers’ unfamiliarity with animal welfare certifications, which may vary depending on the level of awareness in different countries or regions.

Hypothesis H5 was supported, indicating that consumers’ attitude towards animal welfare significantly impacted their behavioral intentions. The findings presented here align with the studies conducted by Alonso et al. [4], as well as Carnovale et al. [55]. This underlines the increasing significance that contemporary consumers place on the well-being of animals in the farming industry and their consideration of relevant certifications. Morales et al. [70] conducted a study in Chile that revealed a clear preference among consumers for products from welfare-respecting production systems, aligning with sustainable egg production. The results of our research correspond to this, showing that consumers in Taiwan understand the principles of animal welfare. They show genuine concern for the well-being of animals that lay eggs, which is in line with studies carried out in Europe and the United States [4]. Sass et al. [71] also found a connection between free-range eggs and consumers associated with rugged personality traits, dietary health, and animal welfare concerns. This aligns with our study, where consumer attitudes towards animal welfare significantly influence their intentions to purchase animal welfare eggs.

Recently, an increasing number of people have begun adopting vegetarian diets motivated by factors such as environmental preservation, reducing carbon emissions, and improving personal health. In contrast, this study’s results suggest that vegetarian individuals’ intent to buy animal welfare-friendly products does not coincide with the observed increase. This finding contradicts the proposition put forward by Ploll and Stern [72], who suggested that individuals with stricter dietary restrictions exhibit more pronounced behaviors related to animal welfare and environmental protection. The disparities in the findings may be attributed to variations in the motivations of the vegetarian participants in our study compared to those examined by Ploll and Stern [72]. These motivations may encompass a range of factors, including health, the environment, and animal welfare, as discussed by Hopwood et al. [73].

Furthermore, despite the rise in the number of pet owners due to declining birth rates, the study revealed that there was no corresponding increase in the intention to purchase animal welfare-friendly products among pet owners. This aligns with the results of Sarial and Bozkurt [74], suggesting that although pet owners generally treat their pets well, they are unwilling to incur additional costs to purchase food that aligns with animal welfare considerations.

Regarding religious beliefs, the findings did not reveal a clear correlation, which is consistent with the findings of Hameed et al. [75]. There was no moderating effect of inherent religious orientation on the relationship between trust in green products and attitudes towards them. In essence, the research suggests that factors such as vegetarianism, having pets, and religious beliefs do not consistently influence a customer’s tendency to purchase products that support animal welfare.

## 6. Concluding Remarks, Constraints, and Prospects for Future Study

This research provides the ensuing conclusions and suggestions derived from the outcomes of the model’s validation.

### 6.1. Research Conclusions

The findings showed that consumers prioritize trust in labels and animal welfare when buying cage-free eggs. The limited knowledge in Taiwan about products that promote animal welfare and their associated labels highlights the necessity of increasing public awareness of these items. There is a rising consumer consciousness about animal welfare, sustainable farming, and eco-friendly consumption, suggesting the increased popularity of animal welfare-friendly products. Efforts should focus on educating consumers about these products to encourage their consumption.

The “animal-friendly livestock production” label in Taiwan lacks specific certification criteria, causing confusion among consumers. The government should promote certified labels and enforce strict regulations to enhance consumer understanding and confidence in selecting animal-friendly products.

By popularizing animal welfare-friendly products, raising awareness, and enforcing clear certification criteria, Taiwan can encourage ethical and sustainable consumer choices, satisfying the demand for animal welfare-friendly options, and promoting a responsible approach to agriculture.

### 6.2. Managerial Implications

In terms of managerial practices, businesses can formulate marketing strategies targeting green values and ethical beliefs to influence and reshape consumers’ existing values. This approach can effectively enhance consumers’ attitudes towards animal welfare-friendly products and stimulate their purchasing behavior. Moreover, professionals in the food and livestock industry should strive to improve animal welfare-friendly practices and promote the availability of such products. Actively pursuing relevant certifications for animal welfare-friendly products is also recommended. Additionally, the government ought to take a proactive role in advocating certified labels and enforcing stringent regulations to ensure the production of animal welfare-friendly products. This will effectively communicate the significance of animal-friendly rearing practices and the production of such products.

### 6.3. Study Limitations and Recommendations for Future Research

One of the limitations of this research is its narrow demographic focus, which might not wholly represent the diverse social classes of the respondents. The study primarily targets those who have purchased or consumed cage-free eggs, therefore missing insights from other groups. Additionally, our demographic analysis predominantly dealt with broad categories, without considering the impact that variables such as age, education level, income, and occupation might have on the study’s results, hence lacking the detailed insights these factors might provide. To address this limitation and better generalize our findings, it will be beneficial to broaden the participant pool in future research. This can include participants who do not purchase or consume animal welfare eggs. Furthermore, a more in-depth analysis considering individual demographic factors will allow us to delve into nuanced understandings. Incorporating these strategies in future research can not only bridge the existing gap but also enhance the breadth and validity of our research.

To enhance future research, our investigation plans to examine different levels of trust and welfare (high vs. low) in relation to consumer behavior, attitudes, and preferences towards cage-free eggs. The main objective of this thorough research is to attain a more profound understanding of the elements that impact consumer choices, and their overall views on free-range eggs.

Furthermore, given the price difference between conventional eggs and cage-free eggs in the market, future research could benefit greatly from investigating how much consumers are prepared to spend on cage-free eggs. Methods such as stated preference, choice experiments, contingent valuation, or revealed preference can be employed to facilitate a more comprehensive analysis of consumer preferences for animal welfare-friendly products.

Finally, consumer attitudes, habits, and preferences towards animal welfare-friendly products vary across different countries. Therefore, it is worthwhile to investigate ways to formulate relevant marketing strategies and policies in response to these cross-country differences.

## Figures and Tables

**Table 1:** Demographic analysis examines the characteristics of a population.

N = 341	Item	Population	Percentage (%)
Gender	Male	164	48.1%
	Female	177	51.9%
Age	20 years and below	29	8.5%
	21–30 years	127	37.2%
	31–40 years	43	12.6%
	41–50 years	68	19.9%
	51–60 years	48	14.1%
	60 years and above	26	7.6%
Level of Education	Middle school or below	17	5.0%
	High school/vocational	78	22.9%
	College/university	206	60.4%
	Master’s or above	40	11.7%
Monthly personal income	Less than NTD 20,000 (USD 660) (inclusive)	75	22.0%
	NTD 20,001–40,000 (USD 660–1320)	127	37.2%
	NTD 40,001–60,000 (USD 1320–1980)	83	24.3%
	NTD 60,001–80,000 (USD 1980–2640)	32	9.4%
	Above NTD 80,001 (USD 2640)	24	7.0%
Occupation	Student	61	17.9%
	Army, civil service, and education	36	10.6%
	Service industry	66	19.4%
	Freelance	29	8.5%
	Traditional manufacturing	39	11.4%
	Specialized occupation (e.g., doctor and lawyer)	18	5.3%
	Other	92	27.0%
Pet ownership	Yes	125	36.7%
	No	216	63.3%
Dietary habits	Omnivorous	318	93.3%
	Vegan	1	0.3%
	Ovo-vegetarian	4	1.2%
	Lacto-vegetarian	1	0.3%
	Lacto-ovo vegetarian	8	2.3%
	Bodhi vegetarian	9	2.6%
Religious belief	Non-religious	143	41.9%
	Folk religion	41	12.0%
	Buddhism	77	22.6%
	Taoism	55	16.1%
	Catholicism	2	0.6%
	Christianity	17	5.0%
	Other	6	1.8%

Note: The New Taiwan Dollar is represented by the abbreviation NTD. The current exchange rate is 1 NTD = 0.033 USD.

**Table 2:** Outcomes regarding factor loading, reliability, and validity.

Variables		Items	Standardized Factor Loadings	CR	AVE	Cronbach’s α
Values	Green values	1. I consider cage-free eggs to be environmentally friendly products.	0.714 ***	0.783	0.644	0.881
		2. I think buying cage-free eggs helps the environment stay sustainable by reducing carbon emissions.	0.762 ***			
		3. I consider cage-free eggs more eco-friendly than eggs from other farming methods.	0.802 ***			
		4. I believe that cage-free eggs meet my expectations for promoting environmental sustainability.	0.792 ***			
	Ethical values	1. I consider it morally correct to purchase cage-free eggs.	0.816 ***			
		2. I would feel guilty if I purchased eggs from inhumane farming practices.	0.837 ***			
		3. I believe that purchasing cage-free eggs is aligned with my principles.	0.803 ***			
Attitude towards cage-free eggs		1. I am very interested in cage-free eggs.	0.885 ***	0.864	0.681	0.857
		2. I believe cage-free eggs are beneficial to me.	0.815 ***			
		3. I would like to learn more about cage-free eggs.	0.771 ***			
Behavioral intention		1. I will prioritize purchasing cage-free eggs.	0.896 ***	0.942	0.802	0.941
		2. I would tell my family and friends about the advantages and disadvantages of cage-free eggs.	0.852 ***			
		3. I will recommend cage-free eggs to others.	0.921 ***			
		4. In the future, I intend to frequently purchase cage-free eggs.	0.912 ***			
Human–nature connectedness		1. I believe humans are an integral part of nature.	0.791 ***	0.875	0.699	0.872
		2. I think humans depend on the natural environment to survive.	0.863 ***			
		3. I think that safeguarding the environment is vital for future generations.	0.853 ***			
Trust in labels		1. I trust that cage-free eggs with certified labels offer better quality assurance.	0.868 ***	0.932	0.774	0.930
		2. I find peace of mind in knowing that eggs are produced on farms with humane management practices.	0.904 ***			
		3. I consider farms with certified labels for cage-free eggs to be trustworthy.	0.919 ***			
		4. I believe that the traceability of certified cage-free eggs ensures the accountability for any issues.	0.824 ***			
Attitude towards animal welfare		1. Valuing animal welfare makes me feel good.	0.846 ***	0.945	0.711	0.945
		2. I believe that animal welfare is essential to the environment.	0.891 ***			
		3. I believe that animal welfare is important for food safety.	0.873 ***			
		4. I think economic animals should be treated better.	0.784 ***			
		5. I think animal welfare organizations are fundamental in ensuring adequate care for animals used in economic activities.	0.834 ***			
		6. I think farms that raise economic animals should receive certification from animal welfare organizations.	0.853 ***			
		7. I think legislation should be in place to ensure adequate welfare for economic animals.	0.818 ***			

*** The *p*-value is less than 0.001.

**Table 3:** Correlation coefficients alongside the square root of AVE.

	Mean	Standard Deviation	1	2	3	4	5	6
1. Values	4.9355	1.20748	**0.802**					
2. Attitude towards cage-free eggs	4.7312	1.31074	0.736 **	**0.825**				
3. Behavioral intention	4.8387	1.40522	0.718 **	0.764 **	**0.896**			
4. Human–nature connectedness	6.0655	1.23799	0.547 **	0.446 **	0.389 **	**0.836**		
5. Trust in labels	5.5696	1.25122	0.731 **	0.699 **	0.697 **	0.562 **	**0.880**	
6. Attitude towards animal welfare	5.6267	1.23982	0.741 **	0.688 **	0.711 **	0.636 **	0.804 **	**0.843**

Note 1: The bold values on the diagonal represent the square root of the AVE for each variable. Note 2: ** The *p*-value is less than 0.05.

**Table 4:** Findings from the Path Analysis and Validations of Hypotheses.

Hypothesized Paths	Unstandardized Coefficient	S.E.	C.R.	*p*	Standardized Coefficients	β	Verification Results
H1: Values → Attitude towards cage-free eggs	2.010	0.301	6.680	***	0.895	0.736	Supported
H2: Attitude towards cage-free eggs → Behavioral intention	0.555	0.084	6.590	***	0.702	0.764	Supported
H3: Human–nature connectedness → Behavioral intention	−0.320	0.093	−3.426	***	−0.180	0.389	Unsupported
H4: Trust in labels → Behavioral intention	0.336	0.122	2.754	**	0.190	0.697	Supported
H5: Attitude towards animal welfare → Behavioral intention	0.614	0.134	4.591	***	0.346	0.711	Supported

Note: *** *p*-value is under 0.001; ** *p*-value is under 0.05.

## Data Availability

The data that support the findings of this study are available from the corresponding author, H.-S.C., upon reasonable request.
